# Smoking cessation can improve quality of life among COPD patients: Validation of the clinical COPD questionnaire into Greek

**DOI:** 10.1186/1471-2466-11-13

**Published:** 2011-02-25

**Authors:** George Papadopoulos, Constantine I Vardavas, Maria Limperi, Apostolos Linardis, George Georgoudis, Panagiotis Behrakis

**Affiliations:** 1 Laboratory of Physiology, School of Medicine, University of Athens, Greece; 2 Smoking and Lung Cancer Research Center, Hellenic Cancer Society, Athens, Greece; 3 National Centre of Social Research, Athens, Greece; 4 School of Physiotherapy, Technological Educational Institute of Athens, Greece

## Abstract

**Background:**

Chronic obstructive pulmonary disease (COPD) remains a major public health problem that affects the quality of life of patients, however smoking cessation may emeliorate the functional effects of COPD and alter patient quality of life.

**Objective-design:**

The aim of this study was to validate the Clinical COPD Questionnaire (CCQ) into Greek and with such to evaluate the quality of life in patients with different stages of COPD, as also assess their quality of life before and after smoking cessation.

**Results:**

The internal validity of questionnaire was high (Cronbach's a = 0.92). The reliability of equivalent types in 16 stabilized patients also was high (ICC = 0.99). In general the domains within the CCQ were strongly correlated with each other, while each domain in separate was strongly correlated with the overall CCQ score (r^2 ^= 0.953, r^2 ^= 0.915 and r^2 ^= 0.842 in regards to the functional, symptomatic and mental domain, respectively). The CCQ scores were also correlated with FEV_1, _(r^2 ^= -0.252, p < 0.001), FEV_1_/FVC, (r^2 ^= -0.135, p < 0.001) as also with the quality of life questionnaire SF-12 (r^2 ^= -0.384, p < 0.001). Smoking cessation also lead to a significant reduction in CCQ score and increase in the SF-12 score.

**Conclusions:**

The self administered CCQ indicates satisfactory validity, reliability and responsiveness and may be used in clinical practice to assess patient quality of life. Moreover the CCQ indicated the health related quality of life gains attributable to smoking cessation among COPD patients, projecting smoking cessation as a key target in COPD patient management.

## Background

Chronic obstructive pulmonary disease (COPD) is a preventable and treatable disease. Its pulmonary component is characterized by airflow limitation that is not fully reversible. This airflow limitation is usually progressive and associated with an abnormal inflammatory response of the lung to noxious particles or gases, such as those emitted during smoking [1]. COPD has considerable implications on the quality of life of patients and may overload the health care system and increase health care expenditure [1,2]. Moreover at a global level, COPD is a significant public health issue and is the fourth leading cause of illness and mortality worldwide, while up to the year 2030 it is expected to be the third largest cause of death [3-5].

Tobacco smoking continues to be a major cause of COPD, while passive smoking may also contribute to respiratory symptoms and COPD progression [4,6,7]. Cigarette smokers have a higher prevalence of respiratory symptoms and lung function abnormalities, while on the contrary, smoking cessation has been found to reduce the rapid decline in FEV_1 _[8]. Smoking cessation is the single most effective--and cost effective--way to reduce the possibility of developing COPD as smoking cessation can prevent or delay the development of airflow limitation, or reduce its progression [9]. With the above in mind, active intervention to help patients quit smoking is a primary tool within the ideal management of COPD. Moreover, the Initiative for Chronic Obstructive Lung Disease includes goals related to clinical (prevention of disease progression and minimization of symptoms) and health-related quality of life (improved exercise tolerance and emotional function) among COPD patients indicating the interaction between respiratory illness and quality of life [10]. Health status, functional status, and quality of life are three concepts often used interchangeably to refer to this same domain of health. Indeed, Health-Related Quality of Life (HRQL) is important for measuring the impact of chronic disease such as COPD. With the above initiative in mind, the necessity to develop new questionnaires that assess quality of life within COPD patients was emphasized so as to help determine and treat functional problems which are important in patients with COPD [11,12]. However as the tool must be easy to use in clinical practice as also evaluate symptoms and the functional and intellectual situation of patients, the Clinical COPD Questionnaire (CCQ) was developed [13]. With the above in mind the aim of the present study was to i) assess the CCQ's validity among both healthy adults and patients with COPD, ii) to assess the test-retest reliability of the CCQ and iii) to assess quality of life among COPD patients before and after smoking cessation.

## Methods

### Subjects

**Figure 1:**
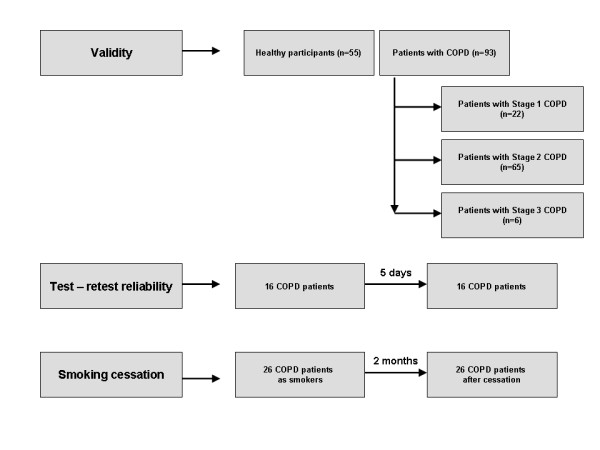
**Flowchart of participant followup**.

Three study populations were recruited so as to evaluate the three separate objectives as depicted in the flowchart of Figure 1. All subjects visited the 10^th ^outpatient pulmonary clinic of the Sotiria hospital in Athens after a scheduled appointment. So as to assess the internal validity and consistency of the CCQ we enrolled 148 subjects (102 men and 46 women), of these 93 had COPD (67 men and 26 women, aged from 41-90 years) and 55 were healthy participants (36 men and 19 women, aged from 41-60 years old). Healthy subjects were hospital staff while none of these controls reported current respiratory symptoms (cough or dyspnoia). All recruited patients with COPD were stabilized and had a history of smoking >12 pack/years. We excluded COPD patients with: 1) significant improvement of FEV_1 _>15% and/or 200 ml compared with baseline (it is diagnostic for bronchial asthma), 2) asthma, chronic heart failure, obstructive sleep apnoea syndrome, cancer or other disabling diseases except COPD, 3) disease exacerbation in the previous four weeks 4) significant self reported shortness of breath in the past 4 days, 5) patients under 40 years of age.

According to the guidelines, COPD was defined by the presence of chronic cough, sputum production and/or dyspnoea and subsequently categorised as either mild, moderate, severe or very severe [10]. According to their current COPD status, the study participants were categorized as: Stage I Mild COPD: 22 patients, 17 men (18.2%) and 5 women (5.37%), Stage-II Moderate COPD: 65 patients, 46 men (49.4%) and 19 women (20.4%), Stage III Severe COPD: 6 patients, 4 men (54.3%) and 2 women (2.15%). Table 1 depicts the characteristics of the study population.

**Table 1:** Descriptive statistics of the study population

		COPD group			
		
	Control group (n = 55)	All stages of COPD (n = 93)	Stage I: Mild COPD (n = 22)	Stage II: Moderate COPD (n = 65)	Stage III: Severe COPD (n = 6)
	**% (n)**	**% (n)**	**% (n)**	**% (n)**	**% (n)**

**Gender(male)**	63.6% (35)	72% (67)	81.8% (18)	69.2% (45)	66.7% (4)

	**Mean ± SD**	**Mean ± SD**	**Mean ± SD**	**Mean ± SD**	**Mean ± SD**

**Age (mean)**	60.45 ± 11.46	66.35 ± 9.68	55.14 ± 7.03	68.80 ± 6.57	81 ± 8.65
**CCQ Total Score**	0.05 ± 0.11	1.437 ± 1.19	1.09 ± 1.17	1.58 ± 1.19	1.17 ± 1.20
**CCQ Symptoms**	0.05 ± 0.19	1.357 ± 1.16	1.15 ± 1.07	1.44 ± 1.20	1.25 ± 1.20
**CCQ Functional state**	0.05 ± 0.10	1.720 ± 1.49	1.25 ± 1.47	1.92 ± 1.48	1.25 ± 1.46
**CCQ Mental state**	0.05 ± 0.16	1.032 ± 1.18	0.66 ± 1.24	1.18 ± 1.17	0.83 ± 0.88
**FEV_1_**	97.65 ± 8.70	70.06 ± 11.1	82.66 ± 2.95	67.94 ± 7.99	46.83 ± 3.93
**FEV1/FVC**	89.42 ± 7.85	62.90 ± 7.7	69.43 ± 2.17	60.75 ± 7.75	62.27 ± 7.63
**SF-12**	3.10 ± 0.33	2.909 ± 0.36	3.14 ± 0.33	2.87 ± 0.33	2.49 ± 0.26

### Lung function testing

All subjects individually, gave their written informed consent for baseline spirometry and before the administration of the questionnaire, as approved by the Medical Ethics Committee of the University of Athens.

Lung function (using FEV_1 _and FVC) was measured according to ERS guidelines with the use of wedge spirometry (ZAN 300 CO diffusion), in base condition in all subjects and 15 minutes metered inhalation of 400 mcg of salbutamol, only in patients with COPD. Spirometry was undertaken on all patients to make a confident diagnosis of COPD and to exclude other diagnoses that may present similar symptoms. Spirometry measurements were evaluated by comparison with reference values based on age, height, sex, and race [14,15].

### Questionnaire selection and procedures

The subjects were requested to complete the Greek version of the SF-12v2 (Short Form Health Survey SF-12v2) and the clinical COPD questionnaire (CCQ) [16] (Table 2). Permission for use was obtained from T. van der Molen (2005), who provided the research team with a translated-back translated version of the CCQ in Greek. Participants completed the questionnaire set in a random order to avoid bias. The administrator used a standardized script to explain the requirements of the questionnaires, and any questions were answered. Test-retest reliability was estimated in a second subgroup of 16 stabilized COPD patients, with the CCQ administered at two separate occasions with a difference of 5 days. Responsiveness of the CCQ after smoking cessation, was examined among a third group of 26 patients (16 men and 10 women) who were while re-administered the CCQ and SF-12v2 after a period of two months after they were counseled to stop smoking and successfully quit (without them taking any medication or therapy to quit).

**Table 2:** The Greek version of the Clinical COPD Questionnaire

ΚΛΙΝΙΚΟ ΕΡΩΤΗΜΑΤΟΛΟΓΙΟ ΓΙΑ ΤΗΝ Χ.Α.Π. Παρακαλούμε βάλτε σε κύκλο τον αριθμό της απάντησης που περιγράφει καλύτερα το πώς νιώθατε κατά την τελευταία εβδομάδα. (μόνο μία απάντηση για κάθε ερώτηση)							
Κατά μέσο όρο, κατά την τελευταία εβδομάδα, πόσο συχνά νιώσατε:	Ποτέ	Σχεδόν ποτέ	Μερικές φορές	Αρκετές φορές	Πολλές φορές	Πάρα πολλές φορές	Σχεδόν συνέχεια
1. **Λαχάνιασμα όταν ήσαστε σε ανάπαυση**;	0	1	2	3	4	5	6
2. **Λαχάνιασμα όταν είχατε σωματικές δραστηριότητες**;	0	1	2	3	4	5	6
3. **Ανήσυχος/η **μήπως κολλήσετε κάποιο κρυολόγημα ή μήπως χειροτερέψει η αναπνοή σας;	0	1	2	3	4	5	6
4. **Θλιμμένος/η (λυπημένος/η) **λόγω των αναπνευστικών σας προβλημάτων; Γενικά κατά την τελευταία εβδομάδα, πόσο από τον χρόνο:	0	1	2	3	4	5	6
5. **Βήχατε;**	0	1	2	3	4	5	6
6. **Είχατε φλέματα;** Κατά μέσο όρο, **κατ**ά **την τελευταία εβδομάδα**, πόσο περιοριστήκατε στις παρακάτω δραστηριότητες λόγω των αναπνευστικών σας προβλημάτων;	0 Δεν περιορίστηκα καθόλου	1 Περιορίστηκα πολύ λίγο	2 Περιορίστηκα λίγο	3 Περιορίστηκα μέτρια	4 Περιορίστηκα πολύ	5 Περιορίστηκα πάρα πολύ	6 Περιορίστηκα τελείως ή ανίκανος να τις κάνω
7. **Εντατικές σωματικές δραστηριότητες **(όπως το να ανεβαίνετε σκάλες, το να βιάζεστε, το να κάνετε αθλητισμό);	0	1	2	3	4	5	6
8. **Μέτριες σωματικές δραστηριότητες **(όπως περπάτημα, νοικοκυριό, μεταφορά πραγμάτων);	0	1	2	3	4	5	6
9. **Καθημερινές δραστηριότητες στο σπίτι **(όπως το να ντύνεστε, πλένεστε);	0	1	2	3	4	5	6
10. **Κοινωνικές δραστηριότητες **(όπως το να μιλάτε, το να είστε με παιδιά, ή το να επισκέπτεστε φίλους/συγγενείς);	0	1	2	3	4	5	6

The CCQ is a very short 10 question survey through which patients are requested to recall their experiences during the previous week in regards to their symptoms, functional and mental state. It is self-administered and takes patients approximately 2 minutes to complete. It is subdivided into three domains: symptom related (items 1-2-5-6), functional state related (items 7-8-9-10) and mental state related (items 3-4). Subjects respond to each question on a 7 point scale (ranging between 0 = asymptomatic/no-limitation, to 6 = extremely symptomatic/totally limited). The overall clinical COPD control score and the score of the three domains is calculated by adding all the scores together and dividing the sum by the number of questions.

### Statistical analysis

The descriptive data is presented as mean ± standard deviation for continuous variables and in percentages (n) for categorical. The internal consistency of the CCQ questionnaire was evaluated with the use of the Cronbach's alpha test while the test retest reliability of the CCQ questionnaire was assessed with the use of the interclass correlation coefficient. During the comparison of the CCQ score before and after smoking cessation, all pairs of questions (before and after smoking cessation) as well as the total score (before and after smoking cessation) were assessed with the use of non parametric tests, as the data was skewed. All reported analyses are based on two sided tests, while statistical significance was noted as p < 0.05. Correlations between the different questionnaires were performed and the R^2 ^values were also noted. The statistical package SPSS 18.0 was used to perform the analysis.

## Results

### Internal consistency of the CCQ

The Cronbach's alpha for the Greek CCQ, was calculated at 0.929 for the patients with COPD (n = 93), and similarly in both men (n = 67) and women (n = 26), with a value of 0.932 and 0.926 respectively. Among the entire population, Cronbach's a, was calculated at 0.943 (n = 148).

### Test-retest reliability

Test-retest reliability was estimated twice, in a separate population of 16 stable patients so as to assess the interclass correlation coefficient (ICC). The questionnaire was administered to the patients for the first time (t1) during their initial visit. A repeat administration (t2) after 5 days was chosen, in order to minimize clinical or cognitive changes but also to reduce any chance recall of previous answers. The mean of ICC value of the total CCQ score was 0.990 (0.971 - 0.996). The scores are showed in Table 3, indicating the test-retest reliability of the CCQ in Greek.

**Table 3:** Test re-test reliability of the CCQ taken with a 5 days difference among patients with COPD as indicated through the Inter Correlation Coefficient (ICC) (n = 16)

		95% C.I	
	ICC	Lower Limit	Upper Limit
**CCQ total score before - CCQ total score after**	0.990	0.971	0.996
**On average, during the past 24 hours, how often did you feel**:			
Short breath at rest?	0.977	0.935	0.992
Short of breath doing physical activities?	0.979	0.941	0.993
Concerned about getting a cold or your breathing getting worse?	0.920	0.788	0.971
Depressed (down) because of your breathing problems?	0.967	0.908	0.988
**In general, during the past 24 hours, how much of the time**:			
Did you cough?	0.990	0.972	0.997
Did you produce phlegm?	0.939	0.836	0.978
**On average, during the past 24 hours, how limited were you in these activities because of your breathing problems**:			
Strenuous physical activities	0.976	0.933	0.992
Moderate physical activities	0.983	0.952	0.994
Daily activities at home	0.948	0.859	0.982
Social activities	0.976	0.932	0.991

### Convergent and divergent validity

In general the domains within the CCQ were strongly correlated with each other, while each domain in separate was strongly correlated with the overall CCQ score (r^2 ^= 0.953, r^2 ^= 0.915 and r^2 ^= 0.842 in regards to the functional, symptomatic and mental domain, respectively). The CCQ scores were also correlated with FEV_1, _(r^2 ^= -0.252, p < 0.001), FEV_1_/FVC, (r^2 ^= -0.135, p < 0.001) as also with the quality of life questionnaire SF-12 (r^2 ^= -0.384, p < 0.001) (Table 4). A statistically significant decrease in breath function tests (FEV_1, _FEV_1_/FVC), and the SF-12 score were noted when the CCQ score increased indicating the construct validity and the external criterion related validity respectively.

**Table 4:** Correlations between questionnaire responses, quality of life and respiratory function in patients with COPD (N = 93)

	CCQ Total	CCQ Symptoms	CCQ Function	CCQ Mental	FEV1	FEV1/FVC	SF
**CCQ Total**	1	0.915**	0.953**	0.842**	-0.252*	-0.135	-0.384**
**CCQ Symptoms**	0.915**	1	0.779**	0.685**	-0.230*	-0.100	-0.292**
**CCQ Function**	0.953**	0.779**	1	0.754**	-0.260	-0.145	-0.435**
**CCQ Mental**	0.842**	0.685**	0.754**	1	-0.164	-0.116	-0.267**
**FEV1**	-0.252*	-0.230*	-0.260*	-0.164	1	0.358**	0.538**
**FEV1/FVC**	-0.135	-0.100	-0.145	-0.116	0.358**	1	0.345**
**SF**	-0.384**	-0.292**	-0.435**	-0.267**	0.538**	0.345**	1

* indicates a statistically significant association of p < 0.05

** indicates a statistically significant association of p < 0.001

### Smoking cessation: Sensitivity-responsiveness of the CCQ and quality of life

Sensitivity responsiveness was examined in a separate population of 26 patients with COPD (16 men and 10 women, with a median age of 66.5 years (range 45-88), that smoked and were recommended to quit smoking. Among these patients that had successfully quit smoking after 2 months, the CCQ was re-administered. The CCQ scores significantly improved after two months of smoking cessation in patients with COPD. Specifically there was a statistically significant decrease in the frequency of developing shortness of breath during physical activities (p = 0.008), in the appearance of cough (p = 0.002) and phlegm (p = 0.008) as also among daily activities at home (p = 0.034). Strenuous physical activity and moderate physical activity were not affected among these patients. It is interesting to note that the frequency of reporting ever being "concerned about getting a cold or your breathing getting worse" as also feeling "depressed because of your breathing problems" was found to decrease statistically significantly after smoking cessation (p = 0.005 and p = 0.011 respectively), as depicted in Table 5.

**Table 5:** Comparison between the CCQ scores before and after smoking cessation among COPD patients (n = 26)

		Before	After			
	N	Mean ± SD	Mean ± SD	p-value^1^	ICC	95% C.I
CCQ total scores before and after	26	1.08 ± 0.82	0.72 ± 0.69	<0.001	0.848	0.690 - 0.929
**On average, during the past 24 hours, how often did you feel**:						
Short breath at rest	26	0.50 ± 0.86	0.19 ± 0.49	0.054	0.367	-0.016 - 0.656
Short of breath doing physical activities?	26	0.88 ± 1.10	0.50 ± 0.86	0.008	0.793	0.591 - 0.902
Concerned about getting a cold or your breathing getting worse?	26	0.88 ± 1.21	0.30 ± 0.73	0.005	0.595	0.276 - 0.795
Depressed (down) because of your breathing problems?	26	0.65 ± 1.01	0.34 ± 0.79	0.011	0.820	0.638 - 0.915
**In general, during the past 24 hours, how much of the time**:						
Did you cough?	26	1.65 ± 1.44	1.00 ± 1.23	0.002	0.734	0.491 - 0.871
Did you produce phlegm?	26	1.38 ± 1.09	0.92 ± 0.89	0.008	0.711	0.452 - 0.859
**On average, during the past 24 hours, how limited were you in these activities because of your breathing problems**:						
Strenuous physical activities	26	2.53 ± 1.88	2.19 ± 1.38	0.083	0.825	0.648 - 0.918
Moderate physical activities	26	1.46 ± 1.83	1.19 ± 1.60	0.161	0.844	0.683 - 0.927
Daily activities at home	26	0.61 ± 1.09	0.38 ± 0.94	0.034	0.874	0.738 - 0.941
Social activities	26	0.23 ± 0.58	0.19 ± 0.56	0.317	0.942	0.876 - 0.974

1: Non parametric tests were applied.

This subgroup of 26 patients with COPD that successfully quit smoking and remained non smokers 2 months after the initial contact also completed the Greek version of the SF-12v2 (before and after two months smoking cessation) through which significant differences in quality of life was noted for all domains (p < 0.05 in all cases) indicating the usefulness of smoking cessation in improving quality of life among COPD patients.

## Discussion

### Main findings

Within the context of this study, smoking cessation among COPD patients was shown to lead within 2 months to an increase in the rated quality of life in a significant number of health domains, as examined with the use of the clinical COPD questionnaire (CCQ) and the SF12. Moreover the CCQ, was identified as a sensitive and useful tool for assessing quality of life among COPD patients.

In regards to the Greek translation, the internal validity of the applied questionnaire was high among both patients with COPD and healthy volunteers, while the elevated correlations between the CCQ's domains indicate a homogenized questionnaire. In comparison to the Dutch, Italian and Swedish versions of the current questionnaire our Cronbach a score was slightly higher indicating its successful translation from English. (T.Van der Molen 2003: a = 0.91, Salvatore Damato 2005: a = 0.89, Björn Ställberg 2009: a = 0.82) [13,17,18].

Chronic obstructive pulmonary disease is a major cause of morbidity and mortality worldwide [1,2]. Among patients with COPD the goals of clinical control include: improved exercise tolerance, emotional function, prevention of disease progression and minimization of symptoms, all of which may lead to an increased quality of life [10]. Quality of life is an important factor in patient treatment and its scientific documentation is a necessity in clinical practice, for instance HRQL in patients with COPD is related to impaired FEV_1 _[19]. With this in mind, the CCQ as seen through the context of this study, can pick up the differences noted between each state of COPD and thus help categorize each patient and assess their symptomatic, mental and functional status, with the latter found to be related to the clinical outcome of lung function tests. In general patients with mild COPD have better respiratory function test (FEV_1_, FEV_1_/FVC) than patients with moderate COPD, and this association was also indicated through our study [20,21].

Among the factors that are leading causes of COPD is tobacco smoking [2,3,8 ]. It causes airflow limitation which is slowly progressive and irreversible. Persistent smoking is associated with rapid longitudinal decline in FEV_1 _and poor prognosis, indicating that [1,4,8,9], smoking cessation is an effective way to reduce the progress of the disease as also the likelihood of actually developing COPD [4,8,9,22]. Smoking cessation leads to prolonged improvements in endothelial function [23] and also associated with substantially improved cardiovascular function and reduced risk of primary and secondary cardiovascular morbidity and mortality [24]. Chronic exposure to cigarette smoke causes increased production of metalloproteinases by macrophages and proteolitic enzymes by neutrophils [25] and decrease in pulmonary mature dendritic cells [26]. Moreover, tobacco smoke compromises the anti-bacterial function of leukocytes, including neutrophils, monocytes, T cells and B cells, providing a mechanistic explanation for increased infection risk which could have an effect on health outcomes [27]. Indeed, patients with COPD have an increased risk of mortality compared to those who do not, with consequent reduction in life expectancy [28]. Smoking cessation can improve the symptoms that COPD patients often experience (such as dyspnoia and cough) as also indicated among the patients of our study, through which smoking cessation led to a statistically significant reduction in COPD symptoms such as cough and dyspnoia as also an increased physical endurance (such as walking up a flight of stairs) with significant clinical implications for patient outcome.

#### Strengths and limitations

The aim of the current study was to assess the change in HRQL among COPD patients before and after smoking cessation, as also to assess the validity and applicability of the CCQ, therefore we did not investigate into how the 26 COPD patients quit, which was not within the scope of the study and therefore we were not able to assess a success rate in smoking cessation. Among the COPD patients that quit smoking during the duration of the study, respiratory assessment was not available during the post cessation assessment and therefore we were unable to assess the role of smoking cessation on respiratory function with clinical observations, however such scientific evidence is already well known [9,22]. Moreover the sample size could have been even larger for a complete validation study and we must note that during the analysis we classified three groups of patients with COPD, out of which the sample of patients with severe COPD was low (n = 6) and therefore the results might not be indicative of patients with severe COPD, as this small number of patients were relatively more healthy in comparison to the patients (n = 65) with mild COPD. Furthermore, we did not examine patients with very severe COPD and these factors should be taken into account in future studies.

## Conclusions

The clinical COPD questionnaire is the first to have been specifically developed and validated to measure control in patients with COPD. The validation of the questionnaire in Greek and in specific clinical pulmonary disease practice, confirms strong discriminative properties, test-retest reliability and responsiveness. Applying the CCQ within the context of this study, smoking cessation was related to improved HRQL scores, and a reduction in COPD related symptoms, indicating the necessity for active interventions by health professionals to help COPD patients quit smoking as a primary tool for the adequate management of COPD and the patient's quality of life.

## Abbreviations

COPD: Chronic obstructive pulmonary disease; CCQ: Clinical COPD Questionnaire; FEV_1_: Forced Expiratory Volume at 1 sec; FVC: Forced Vital Capacity; HRQL: Health-Related Quality of Life; ERS: European Respiratory Society; SF-12v2: Short Form Health Survey 12 version 2; ICC: Interclass correlation coefficient

## Competing interests

The authors declare that they have no competing interests.

## Authors' contributions

GP carried out the clinical assessments and performed the statistical analysis. CIV had the key role in manuscript preparation and participated in study coordination. CIV, ML an AL participated in the study coordination, statistical analysis, and manuscript preparation. PB conceived of the study, and participated in its design and coordination and helped to draft the manuscript. All authors read and approved the final manuscript.

## Pre-publication history

The pre-publication history for this paper can be accessed here:

http://www.biomedcentral.com/1471-2466/11/13/prepub
